# Supplementary material for: Exome sequencing study of 20 patients with high myopia
Source: PeerJ. 2018 Sep 17;6:e5552. doi: 10.7717/peerj.5552 (PMC6148412; doi:10.7717/peerj.5552)
Supplement: Supplemental Information 1 — Positions are QTL positions on human genome assembly GRCh37. [file peerj-06-5552-s001.docx]

| Cyto Location | Mim Number | QTL name | Genes Identified | Positions | | |
| --- | --- | --- | --- | --- | --- | --- |
|  |  |  |  | Chr | Start | End |
| 1p36 | 610320 | MYP14 |  | 1 | 10000 | 222691127 |
| 1p22.2 | 614159 | MYP21 | ZNF644 | 1 | 91380854 | 91487825 |
| 2q37.1 | 609995 | MYP12 |  | 2 | 230964716 | 235608644 |
| 3q26 | 609257 | MYP8 |  | 3 | 160717788 | 182717788 |
| 4p16.3 | 104225 | MYP23 | LRPAP1 | 4 | 3505323 | 3534223 |
| 4q12 | 609258 | MYP9 |  | 4 | 52666166 | 59366166 |
| 4q22-q27 | 609994 | MYP11 |  | 4 | 88021152 | 123721155 |
| 4q35.1 | 615421 | MYP22 | CCDC111 | 4 | 185570766 | 185616112 |
| 5p15.33-p15.2 | 612554 | MYP16 |  | 5 | 10000 | 15000109 |
| 5p15.1-p13.3 | 613969 | MYP19 |  | 5 | 15000109 | 33800105 |
| 5q31.1 | 600608 | MYP25 | P4HA2 | 5 | 131525839 | 131563555 |
| 7p15 | 608367 | MYP17, MYP4 |  | 7 | 20939619 | 28839617 |
| 8p23 | 609259 | MYP10 |  | 8 | 10000 | 12657509 |
| 10q21.1 | 612717 | MYP15 |  | 10 | 52859760 | 61159758 |
| 11p13 | 609256 | MYP7 |  | 11 | 31021547 | 36421550 |
| 12q13.3 | 608730 | MYP24 | SLC39A5 | 12 | 56623819 | 56631629 |
| 12q21-q23 | 603221 | MYP3 |  | 12 | 71493780 | 108993776 |
| 13q12.12 | 614166 | MYP20 |  | 13 | 23174139 | 25474138 |
| 14q22.1-q24.2 | 255500 | MYP18 |  | 14 | 50866718 | 73766708 |
| 17p13.1 | 160740 | MYPOP | MYH2 | 17 | 10424464 | 10453016 |
| 17q21-q22 | 608474 | MYP5 |  | 17 | 37956253 | 57577361 |
| 18p11.31 | 160700 | MYP2 |  | 18 | 2899998 | 7199998 |
| 22q13.33 | 604272 | MYP6 | SCO2 | 22 | 50961996 | 50964867 |
| Xq23-q27.2 | 300613 | MYP13 |  | X | 24727075 | 142087786 |
| Xq28 | 310460 | MYP1 |  | X | 44864594 | 155260560 |
