# Supplementary figures and images for: Exome sequencing study of 20 patients with high myopia
Source: PeerJ. 2018 Sep 17;6:e5552. doi: 10.7717/peerj.5552 (PMC6148412; doi:10.7717/peerj.5552)

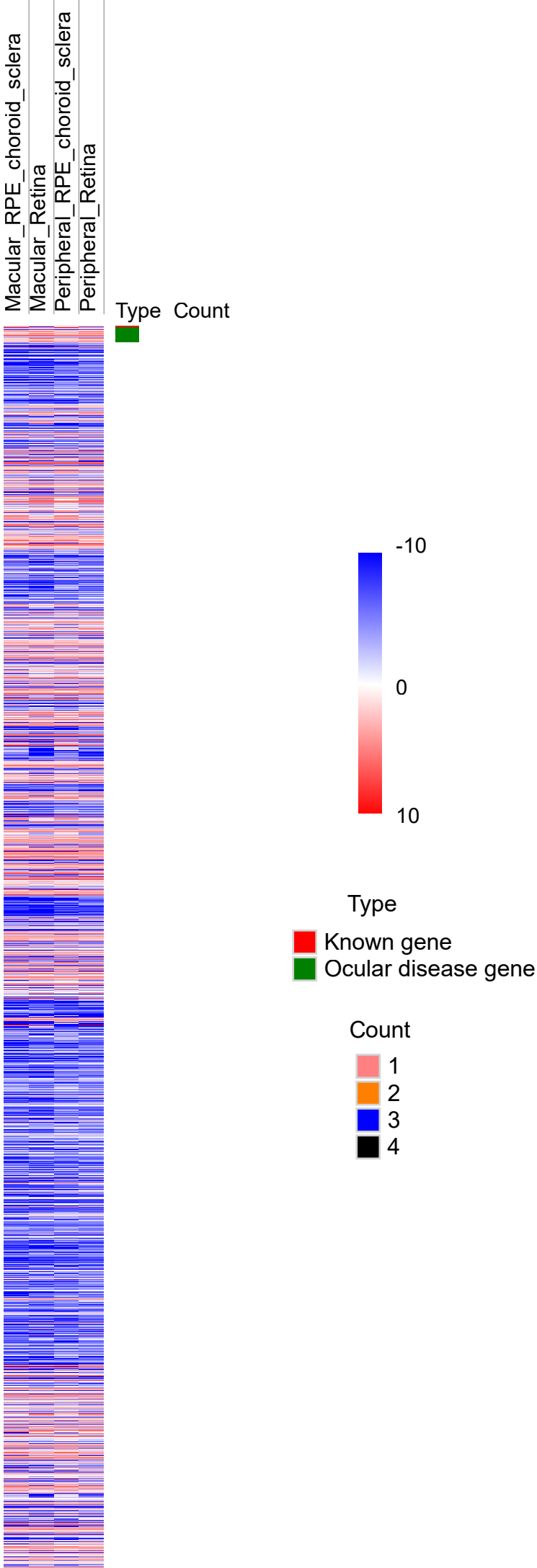

Supplement: Supplemental Information 5 — Compare to other genes in the genome, HM know genes and ocular disease genes are highly expressed in retina, retinal pigment epithelium (RPE), choroid and sclera. The values shown in the heatmap are log2(RPKM+0.001). [file peerj-06-5552-s005.pdf]

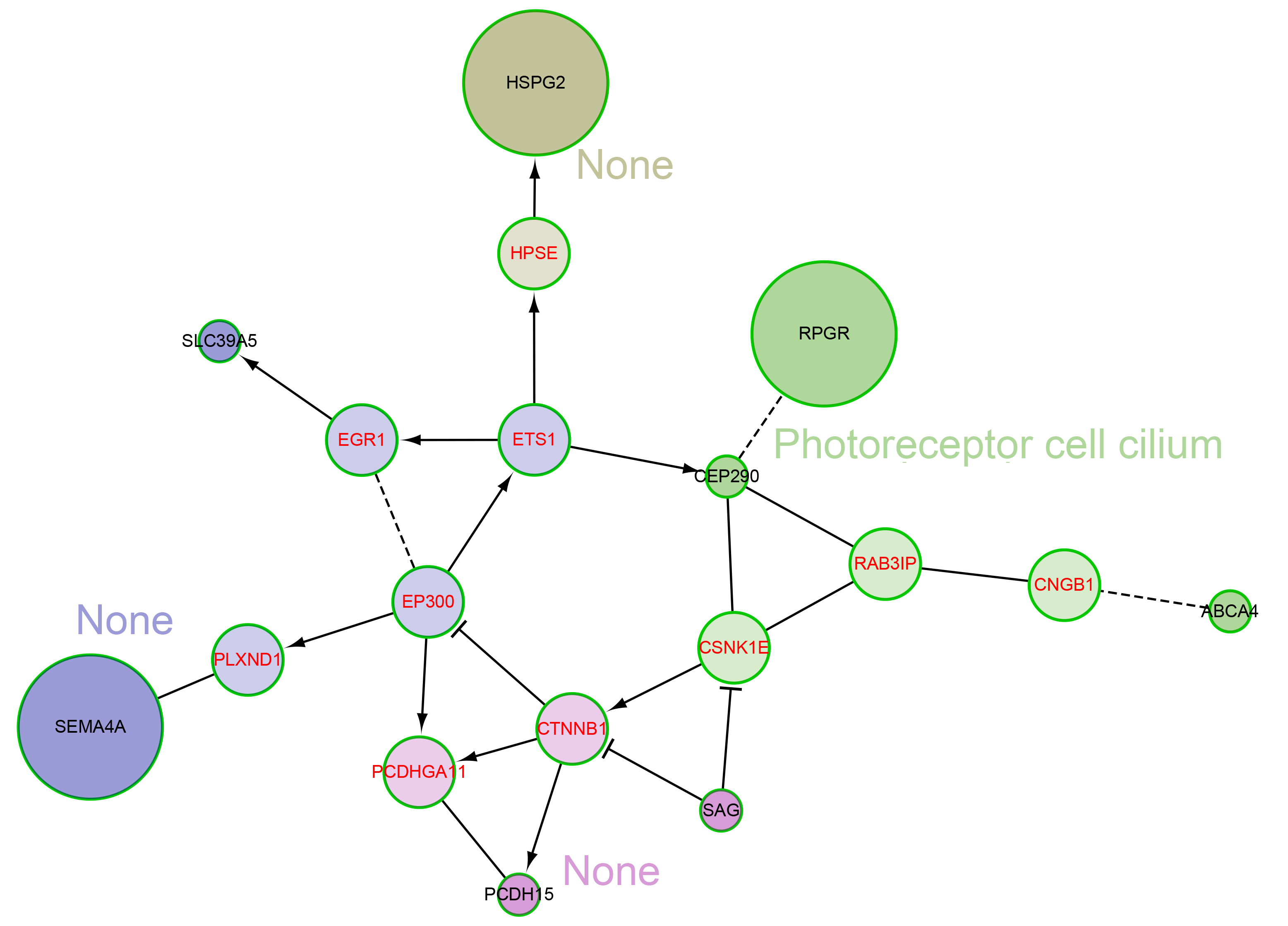

Supplement: Supplemental Information 6 — This network can be divided into 4 network modules. Principal biological process of each module was calculated using the eye disease genes as background instead of the genomic background as in Fig. 4. The rest is the same as in Fig. 4. [file peerj-06-5552-s006.png]
